# 

**Published:** 1857-01

**Authors:** 


### CONTENTS.

### *ORIGINAL COMMUNICATIONS.*

PAGE

ARTICLE I.—Fœticide. An Essay by J. Boring, M. D., Professor Obstetrics, &c., Atlanta Medical College; read before the Atlanta Medical Society, and published by that body,........................................ 257

ARTICLE II.—A Case of Gaseous Colic. By Thos. S. Denny, M. D.,......... 268

### *SELECTIONS.*

Effervescing Powders,........................................................ 269

Experimental and Clinical Researches applied to Physiology and Pathology... 275

Remarks upon the Medicinal Plants of Cherokee, Georgia,........................ 283

Review of Dr. N. S. Davis' Annual Address before the Illinois State Medical Society,........................................................ 286

Cn Inflammation of the Uterus, and Induration and Ulceration of the Neck of that Organ,........................................................ 291

Contributions to the Physiology and Pathology of the Heart,........................ 296

Effect of Belladonna in immediately arresting the Secretion of Milk,............ 300

Ergot of Wheat,........................................................ 302

### *EDITORIAL AND MISCELLANEOUS.*

Atlanta Medical College,........................................................ 303

Editorial Correspondence,........................................................ 304

Introductory Lectures,........................................................ 307

To Delinquents,........................................................ 319

Effect of Carbonic Acid on the Gravid Uterus,........................................ 320

